# Genome-wide analysis of DNA methylation identifies novel differentially methylated regions associated with lipid accumulation improved by ethanol extracts of *Allium tubersosum* and *Capsella bursa-pastoris* in a cell model

**DOI:** 10.1371/journal.pone.0217877

**Published:** 2019-06-06

**Authors:** Moonju Hong, Jin-Taek Hwang, Eun Ju Shin, Haeng Jeon Hur, Keunsoo Kang, Hyo-Kyoung Choi, Min-Yu Chung, Sangwon Chung, Mi Jeong Sung, Jae-Ho Park

**Affiliations:** 1 Division of Food Functionality, Korea Food Research Institute, Iseo-myeon, Wanju-gun, Republic of Korea; 2 Department of Food Biotechnology, University of Science & Technology, Daejeon, Republic of Korea; 3 Department of Microbiology, College of Natural Sciences, Dankook University, Cheonan, Republic of Korea; Barts and The London School of Medicine and Dentistry Blizard Institute, UNITED KINGDOM

## Abstract

Hepatic steatosis is the most common chronic liver disease in Western countries. Both genetic and environmental factors are known as causes of the disease although their underlying mechanisms have not been fully understood. This study investigated the association of DNA methylation with oleic acid-induced hepatic steatosis. It also examined effects of food components on DNA methylation in hepatic steatosis. Genome-wide DNA methylation of oleic acid (OA)-induced lipid accumulation *in vitro* cell model was investigated using reduced representation bisulfite sequencing. Changes of DNA methylation were also analyzed after treatment with food components decreasing OA-induced lipid accumulation in the model. We identified total 81 regions that were hypermethylated by OA but hypomethylated by food components or vice versa. We determined the expression of seven genes proximally located at the selected differentially methylated regions. Expression levels of *WDR27*, *GNAS*, *DOK7*, *MCF2L*, *PRKG1*, and *CMYA5* were significantly different between control vs OA and OA vs treatment with food components. We demonstrated that DNA methylation was associated with expression of genes in the model of hepatic steatosis. We also found that food components reversely changed DNA methylation induced by OA and alleviated lipid accumulation. These results suggest that DNA methylation is one of the mechanisms causing the hepatic steatosis and its regulation by food components provides insights that may prevent or alleviate lipid accumulation.

## Introduction

Nonalcoholic fatty liver disease (NAFLD) is a chronic liver disease caused by fat accumulation in the liver due to imbalance between triglyceride (TG) acquisition and removal without alcohol consumption [1]. Progress of NAFLD ranges from simple hepatic steatosis to non-alcoholic steatohepatitis (NASH), fibrosis, and even hepatic cancer. NAFLD is associated with obesity, dyslipidemia, and insulin resistance, which are also known as characteristics of metabolic syndromes [2]. Although the pathogenesis of NAFLD is not fully understood, it has been shown that hepatic de novo lipogenesis is increased by activation of lipogenic factors such as SREBP-1c, PPARγ, and fatty acid synthase (FASN) [2–5]. Subsequently, accumulation of free fatty acids (FFAs) in the liver causes lipotoxicity and oxidative stress, which lead to hepatocyte injury and progress to NASH and fibrosis [2–4]. It is of interest that dietary factors affect *de novo* hepatic lipogenesis via the crucial factors FASN and PPARγ, and can thereby mitigate NAFLD and obesity, based on a cell and an animal model [6, 7]. However, underlying mechanisms of the regulation have not been clearly elucidated.

Substantial emerging evidence has demonstrated that the development and progression of NAFLD is regulated by epigenetic mechanisms including DNA methylation, histone modification and non-coding RNAs [8–13]. In addition, it was reported that both DNA methylation and histone modification are regulated by dietary factors in animal models [14, 15]. However, the relevance between DNA methylation and histone modification has not been clearly elucidated.

Over the past three decades, it has been shown that various dietary factors, including methyl donors, protein, polyunsaturated fatty acid, sugar, and bioactive components, modulate epigenetic status and affect gene expression in various cell and animal models of human diseases including NAFLD [16, 17].

In animal models of fatty liver, deficiency of methyl-donors such as betaine, choline, and folate affects one-carbon metabolism, and consequently progression to NASH [18, 19]. In high-fat-sucrose diet-induced obesogenic mice, dietary methyl-donor supplements improved fatty liver by regulating DNA methylation of *FASN* and its expression [20]. Consistent with this, Chang et al. showed that berberine modulated DNA methylation of the promoter of microsomal triglyceride transfer protein, which is a key gene in lipid homeostasis [21]. Lingonberries prevent hepatic steatosis through regulation of DNA methylation of genes associated with inflammation and lipid synthesis in a high-fat diet-induced animal model [22]. These show that not only methyl-donors but also dietary components affect DNA methylation in an animal model of fatty liver.

Modification of histones by dietary components is also involved in prevention and/or attenuation of NAFLD. In previous study, it has been demonstrated that hepatic steatosis was improved through inhibition of histone acetylation by extract of *Allium tuberosum* (EAT) containing sulfur and phenolic compounds [23]. In addition, extract of *Capsella bursa-pastoris* (ECB) containing flavonoids decreased lipid accumulation through inhibition of histone acetyltransferase in an *in vitro* cell model [24].These results suggest that dietary components including EAT and ECB may be applicable for reducing lipid accumulation and improving hepatic steatosis. These studies also showed that 200–400 μg/mL EAT or ECB affect lipid accumulation and epigenetic status in HepG2 cells without toxic effects. However, little is known about global effects on DNA methylation by treatment with dietary components EAT or ECB in hepatic steatosis.

In this study, we performed reduced representation bisulfite sequencing (RRBS) to investigate changes in genome-wide DNA methylation by EAT and ECB in an OA-induced hepatic steatosis model. We identified differential methylated regions (DMR) by OA or treatment of EAT and ECB, and showed the regulation of gene expression by the DMRs in the model.

## Materials and methods

### Chemicals, reagents and antibodies

Oil red O (O0625), sodium oleate (O7501), thiazoyl blue tetrazolium bromide (M5655) were purchased from Sigma-Aldrich (St. Louis, MO, USA). Antibody against fatty acid synthase (FASN, C20G5) was purchased from Cell Signaling Technology (Boston, MA, USA). HRP-conjugated goat anti-rabbit IgG (A120-101P) was purchased from Bethyl Laboratories (Montgomery, TX, USA).

### Preparation of *Allium tuberosum* extract and *Capsella bursa-pastoris* extract

*Allium tuberosum* and *Capsella bursa-pastoris* were purchased from a local market (Republic of Korea) and extracted in a 10-fold volume of 70% ethanol by shaking for 24 h at 25°C. Precipitate was then removed by centrifugation at 8000 *g* for 30 min, and supernatants were freeze dried and used.

### Cell culture and treatment of experimental groups

HepG2 (human Caucasian hepatocellular carcinoma) cells were obtained from the American Type Culture Collection (Mannassas, VA, USA). Cells were maintained in high-glucose Dulbecco’s modified Eagle’s medium (DMEM) supplemented with 10% fetal bovine serum (FBS) and 1% antibiotics (including penicillin, streptomycin and amphotericin B), which were purchased from Welgene Inc. (Daegu, Republic of Korea). The cells were maintained at 37°C in a humidified atmosphere of 5% CO_2_. Cells were incubated for 24 h in one of four media: I, 1% BSA-supplemented low-glucose DMEM (control); II, 0.5 mM oleic acid (OA) in 1% BSA-supplemented low-glucose DMEM; III, 0.5 mM OA in 1% BSA-supplemented low-glucose DMEM with 200 μg/mL EAT; IV 0.5 mM OA in 1% BSA-supplemented low-glucose DMEM with 200 μg/mL ECB.

### Cell toxicity

Cells were seeded into a 96-well plate at a density of 8x10^4^ cells/well. HepG2 cells were treated with EAT or ECB at 0, 200, 400, 800 or 1600 μg/mL for 24h. After 24h incubation, 16 μl MTT solution (1000 μg/ml) was added to each well and incubated for 4h. Culture medium was removed, 100 μl of DMSO was added into each well. Absorbance was measured at 540 nm.

### Oil red O staining

HepG2 cells were cultured in a 24-well plate (3x10^5^ cells/well). Then cells were treated with 0.5 mM OA (II), 0.5 mM OA + 200 μg/mL EAT (III) or 200 μg/mL ECB (IV) for 24hr. After washing with 200 μL of phosphate-buffered saline (PBS), cells were fixed with 200 μL of 4% paraformaldehyde for 15 min. Then the cells were washed three times again with PBS and stained with 200 μL of 0.1% Oil red O solution for 10 min. Cells were dried and incubated with 60% isopropanol for 10 min, and absorbance was determined at 510 nm.

### Reduced representation bisulfite sequencing (RRBS) library preparation and sequencing

To construct RRBS libraries with MspI and ApeKI, 500 ng of input genomic DNA in 50 μl was digested with MspI (NEB, Ipswich, MA, USA) at 37°C for 7 h. ApeKI (NEB) was then added and incubation was continued at 75°C for 16–20 h. The digested products were purified with a MiniElute PCR Purification Kit (Qiagen, Venlo, Netherlands). After purification, dA was added to the digested products with blunt-ended ligation, followed by ligation of methylated-adapter. A slice of the 160–420 bp fraction was excised from 2% agarose gel. Bisulfite conversion was conducted using a ZYMO EZ DNA Methylation-Gold Kit (ZYMO Research, Irvine, CA, USA) following the manufacturer’s instructions. The final libraries were generated by PCR amplification using PfuTurbo Cx Hotstart DNA polymerase (Agilent technologies, Santa Clara, CA, USA). RRBS libraries were analyzed by an Agilent 2100 Bioanalyzer (Agilent Technologies). Before sequencing the samples, the quantity of sequenceable library fragments was determined via qPCR. Samples were then diluted to 10 nM with elution buffer (QIAGEN). RRBS libraries were sequenced with a NextSeq500 (Illumina, San Diego, CA, USA) at LAS Inc. (Kimpo, Republic of Korea). The sequence data have been deposited in NGS data of Korea Centers for Diseases Control & Prevention (KCDC) and are accessible through Clinical & Omics Data Archive (CODA) accession number R001414 (http://coda.nih.go.kr/coda/frt/index.do).

### RRBS data analysis

We performed FastQC v0.11.2 (http://www.bioinformatics.babraham.ac.uk/projects/fastqc/) to control the quality of raw reads, and trimmed adaptor sequencing using trim galore v0.4.1 (http://www.bioinformatics.babraham.ac.uk/projects/trim_galore/). Trimmed sequences were aligned to the human reference genome (hg19) using BS-seeker2 v2.0.10 (Guo et al., 2013) with Bowtie2. We built double enzyme MspI (CCGG) and ApeKI (GCWGC) fragments with length range 30–500 bp in silico to cover MspI and ApeKI fragments of RRBS libraries. We aligned the reads with Bowtie2 in local alignment mode allowing four mismatches per read. Unmapped reads were remapped in paired-end mode to improve mapping rates. Where two paired-end mates overlapped, we called methylation levels of each CpG site after removing one mate.

To avoid low mapping efficiency due to adapter contamination in the sequencing data, size selection (160–420 bp) was performed. It was found that mappability (>70%, S2 Table) and depth (>40 x, S2 Table) were better than those observed in previous studies, although these studies analyzed different cells and tissues [25, 26]. This suggested that our sample preparation, generation of DNA methylomes, processes of sequencing, and mapping analysis had no critical problems. However, physical coverage could not be calculated in this analysis because C to T is the most common substitution (~ 65%) in all single nucleotide polymorphisms (SNPs) and could not be distinguished from C to T conversion by bisulfite treatment [25]. In general, less than four million CpGs out of 29 million in the genome were physically covered by our RRBS screening [26].

### Differentially methylated region (DMR) analysis

We used a custom Perl script to identify DMRs (100 bp) between groups. Briefly, DNA methylation levels on the genome were profiled by sliding a fixed-size window (100 bp) in 50 bp increments through the reference genome (hg19). DNA methylation ratios (0 to 1) of all CpG sites in a given window were compared between two groups (control vs OA, OA+EAT vs OA and OA+ECB vs OA) using the Mann-Whitney U test (*p* < 0.01). To filter out unreliable DMR candidates, regions covered by less than 10 reads or showing mean difference of < 0.2 between groups were discarded. Identified DMRs were annotated using HOMER (v5.7) with the UCSC reference gene annotation (hg19).

### Western blot analysis

HepG2 cells were harvested and homogenized in a cell lysis buffer (Cell Signaling Technology, Beverly, MA, USA) containing a Xpert phosphatase and protease inhibitor cocktail solution (GenDEPOT, Barker, TX, USA). Lysates were centrifuged at 10,000 *g* for 15 min at 4°C. Total cellular proteins (20 μg) were loaded on SDS-PAGE and transferred onto nitrocellulose (NC) membranes (GE Healthcare Life Science, Pittsburgh, PA, USA). Blocking buffer contained 5% skim milk in TBS-T at room temperature. Blots were incubated with primary antibody against FASN (Cell Signaling Technology, Beverly, MA, USA) overnight at 4°C. Secondary antibody conjugated with horseradish peroxidase was complexed with primary antibody and developed with an ECL detection kit (DoGEN, Seoul, Korea).

### Quantification of gene expression using real-time PCR

RNA was extract from the treated cells with an RNeasy Mini kit (Qiagen), according to the manufacturer’s instructions. A total of 500 ng RNA was reverse-transcribed with reverse transcriptase (TOYOBO, Osaka, Japan) at 30°C for 10 min, 42°C for 20 min, and 99°C for 5 min. Relative quantification of gene expression was determined with the cDNA and primers listed in S1 Table. The reaction was carried out using SYBR green super mix (TOYOBO) and a thermal cycler (Bio-Rad, Hercules, CA, USA). Amplification conditions consisted of 40 cycles of 95°C for 10 sec, 58°C for 10 sec, 72°C for 20 sec, and a final melting curve step.

### Statistical analysis

All results were shown as the mean ± S.D. Statistical significances between groups were assessed using unpaired t-tests, using GraphPad Prism 5 Software (San Diego, CA, USA). Statistical significance was accepted at *p* < 0.05, *p* < 0.01 and *p* < 0.001.

## Results

### Establishment of the cell model of hepatic steatosis and RRBS analysis

**Fig 1:**
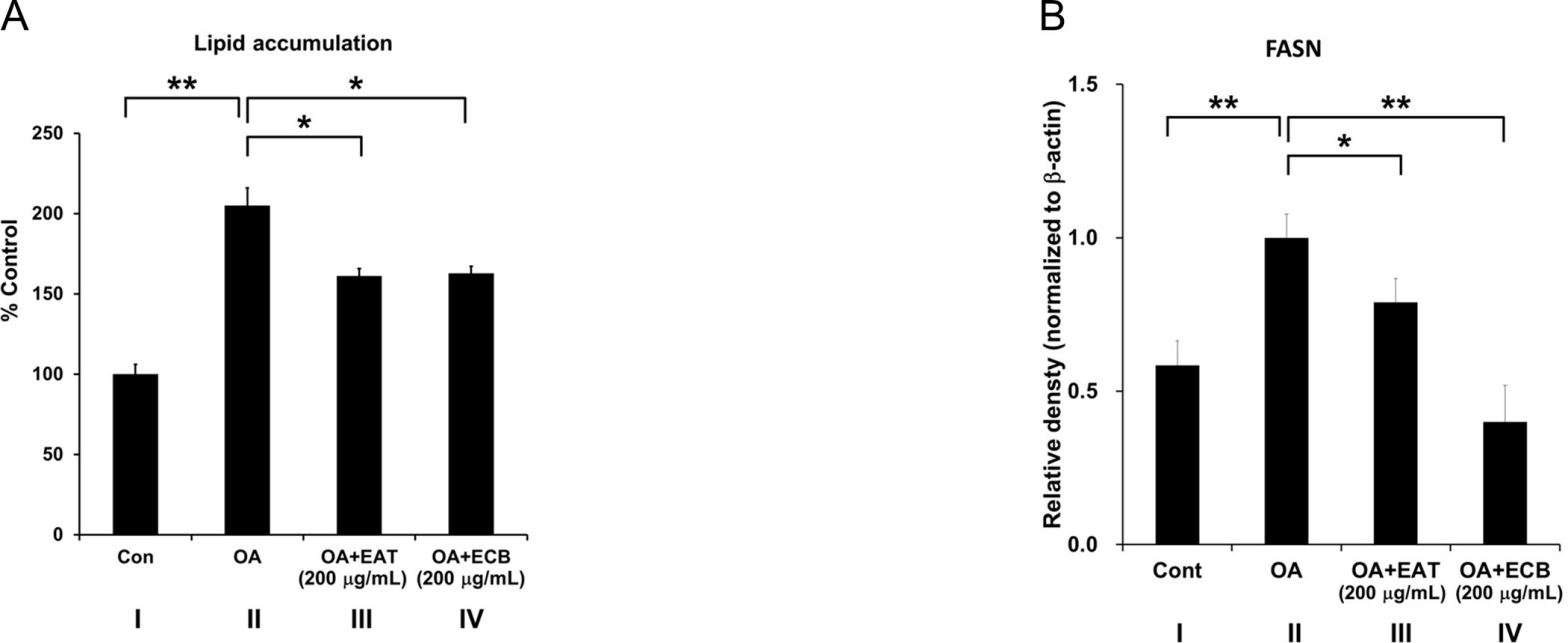
Lipid accumulation and expression of FASN in HepG2 cells. (A) Effects of ECB or EAT treatment on lipid accumulation in OA-induced hepatic steatosis. HepG2 cells were stimulated with 0.5 mM OA (II), OA with EAT (200 μg/mL, III) and OA with ECB (200 μg/mL, IV) for 24 h. Oil red O dye as fat drops were quantified (n = 3, I and II; n = 4, III and IV). (B) FASN expression was calculated using ImageJ (n = 6, I and II; n = 4, III and IV). Data was shown as mean ± SD. **: *p* < 0.01, *: *p* <0.05.

Consistent with previous reports showing that oleic acid stimulated lipid accumulation in HepG2 cells and increased expression of FASN [23, 24], OA induced lipid accumulation up to 2 times and 200 μg/mL EAT (III) or ECB (IV) decreased the lipid accumulation (Fig 1A). We assessed cytotoxicity of EAT or ECB in HepG2 using MTT assay. Treatment with EAT or ECB (0, 200, 400, 800 μg/mL) did not induced cytotoxicity in HepG2 cells (S1 Fig). Since it is known that FAS is a lipogenic enzyme which regulates fatty acid synthesis [27], we further examined the beneficial effects of EAT and ECB on protein expression change of FASN. Increased FASN expression by OA was significantly attenuated by treatment with EAT or ECB in the hepatic steatosis model (Fig 1B and S2 Fig). This showed that our hepatic cell model system was adequate for further investigation of the underlying mechanisms of hepatic steatosis.

As digestion of genomic DNA with double restriction enzymes has been found to increase CpG coverage [28], MspI and ApeKI, were used in this study to efficiently determine genome-wide DNA methylation. In addition, to decrease the likelihood of false positives, less than ten sequenced reads were excluded from our analysis.

Although emerging data shows that single CpGs can be important in regulation of gene expression [29, 30], roles of single CpGs in gene regulation are still debatable. To avoid selection of single differentially methylated CpGs, averages of all CpGs in 100 bp were calculated and statistically analyzed to identify DMRs. Non-CpGs, CHH and CHG, were excluded from the analysis since the mechanisms underlying whether non-CpG methylation plays a role in gene regulation have not been clearly elucidated [31, 32].

### Genome-wide methylation analysis in a cell model of hepatic steatosis

To investigate the underlying mechanisms of lipid accumulation during hepatic steatosis, global DNA methylation pattern, was analyzed using reduced representation bisulfite sequencing (RRBS). A total of twelve samples (n = 3 for each group) were prepared for RRBS as listed in S2 Table. In total, 517 million reads were sequenced and 378 million of these were mapped to the human reference genome. More than 70% of reads were successfully mapped. Sequencing depth ranged from 42 to 97 reads throughout the reference genome (S2 Table).

### Selection of putative DMRs modulated by EAT and ECB in hepatic steatosis

Without consideration of statistical significance or threshold of changes of DNA methylation level, 29,245 differentially methylated regions (DMRs) between control (I) and OA (II) were identified (Table 1). The numbers of DMRs for OA (II) vs OA+EAT (III) and OA (II) vs OA+ECB (IV) were 31,575 and 18,442, respectively. The genomic regions of DMRs were classified into 11 groups including repeats (34–40%), introns (20–24%), intergenic regions (23–24%), exons (6%), TSSs (4%), CpG islands (3%), transcription termination sites (2%), 3’ UTRs (1%), 5’ UTRs (< 1%), non-coding RNA (< 1%), and not determined (NA, <1%). More than 60% of these DMRs were located in repeats and intergenic region.

**Table 1:** Distribution of DMRs in genomic regions.

	TSS	TTS	Exon	5UTR	3UTR	CpG island	Repeats	Intron	Intergenic	Non-coding	NA	Total
**OA (II) vs Control (I)**	1,094	469	1,709	94	281	857	10,261	6,836	7,149	262	233	29,245
	3.7	1.6	5.8	0.3	1.0	2.9	35.1	23.4	24.4	0.9	0.8	100 (%)
**EAT (III) vs OA (II)**	1,174	546	1,908	126	349	966	10,766	7,592	7,634	307	207	31,575
	3.7	1.7	6.0	0.4	1.1	3.1	34.1	24.0	24.2	1.0	0.7	100 (%)
**ECB (IV) vs OA (II)**	645	344	1,014	74	179	559	7,541	3,638	4,162	148	138	18,442
	3.5	1.9	5.5	0.4	1.0	3.0	40.9	19.7	22.6	0.8	0.5	100 (%)

TSS (transcription start site), TTS (transcription termination site), Repeats (SINE, LINE, Alu, Simple repeats, LTR), NA (not assigned)

To identify significant DMRs, we selected regions where the difference in methylation level was more than 20% between groups (Mann-Whitney test, *p* < 0.01). In the OA group (II) compared to the control group (I), there was a total of 406 DMRs, including 215 hypermethylated and 191 hypomethylated (Table 2). In the OA+EAT-treated group (III) compared to the OA group (II), 532 DMRs were identified, with 296 as hypermethylated and 236 hypomethylated. In the OA+ECB group (IV) compared to the OA group, there was a total of 265 DMRs of which 109 were hypermethylated and 156 were hypomethylated. It is of interest that about 60% of the identified significant DMRs were located in CpG islands (18–20%), exons (15–25%), TSSs (11–13%), and 5’ UTRs (> 1%), and therefore more likely to be involved with gene expression (Table 3).

**Table 2:** Summary of selected significant DMRs.

Difference of methylation at CG > 20% (*p* < 0.01) 100 bp window, Mann-Whitney Test, 10 read cut			
	Hypermethylation	Hypomethylation	Total number
OA (II) vs Control (I)	215	191	406
EAT (III) vs OA (II)	296	236	532
ECB(IV) vs OA (II)	109	156	265

**Table 3:** Distribution of selected significant DMRs among genomic regions.

	TSS	TTS	Exon	5UTR	3UTR	CpG island	Repeats	Intron	Intergenic	Non-coding	Total
OA (II) vs Control (I)	53	17	66	8	4	79	51	81	39	8	406
	13.1	4.2	16.3	2.0	1.0	19.5	12.6	20.0	9.6	2.0	100 (%)
EAT (III) vs OA (II)	71	13	79	4	6	93	107	75	75	9	532
	13.3	2.4	14.8	0.8	1.1	17.5	20.1	14.1	14.1	1.7	100 (%)
ECB (IV) vs OA (II)	30	18	66	3	4	49	34	23	36	2	265
	11.3	6.8	24.9	1.1	1.5	18.5	12.8	8.7	13.6	0.8	100 (%)

TSS (transcription start site), TTS (transcription termination site), Repeats (SINE, LINE, Alu, Simple repeats, LTR)

**Fig 2:**
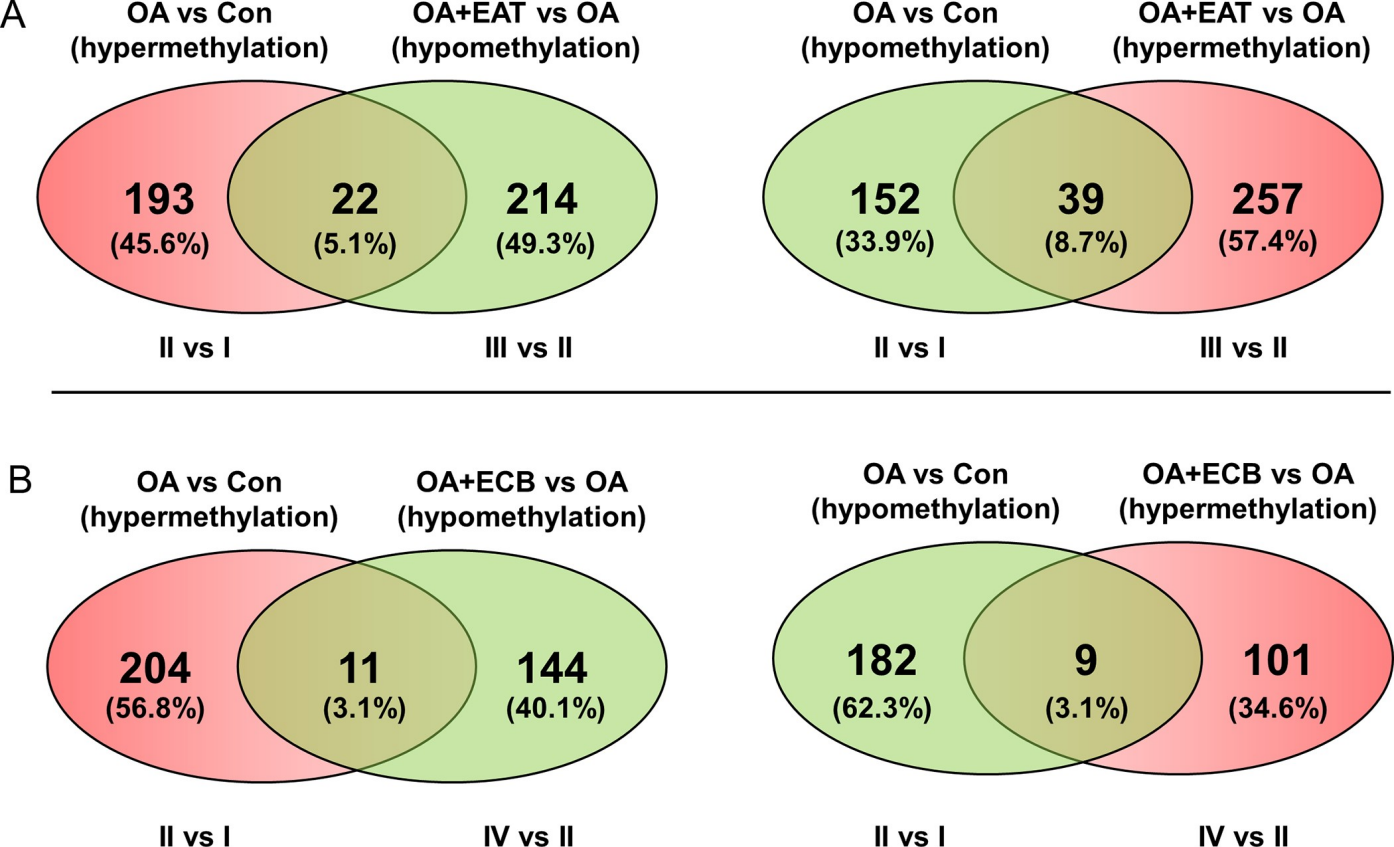
Venn diagram of selected DMRs. (A) Venn diagram showing numbers of DMRs in the OA (II) vs control (I) and OA+EAT (III) vs OA (II) comparisons. (B) Venn diagram showing numbers of DMRs in the OA (II) vs control (I) and OA+ECB (IV) vs OA (II) comparisons. Red and green colors indicated hypermethylation and hypomethylation, respectively.

**Fig 3:**
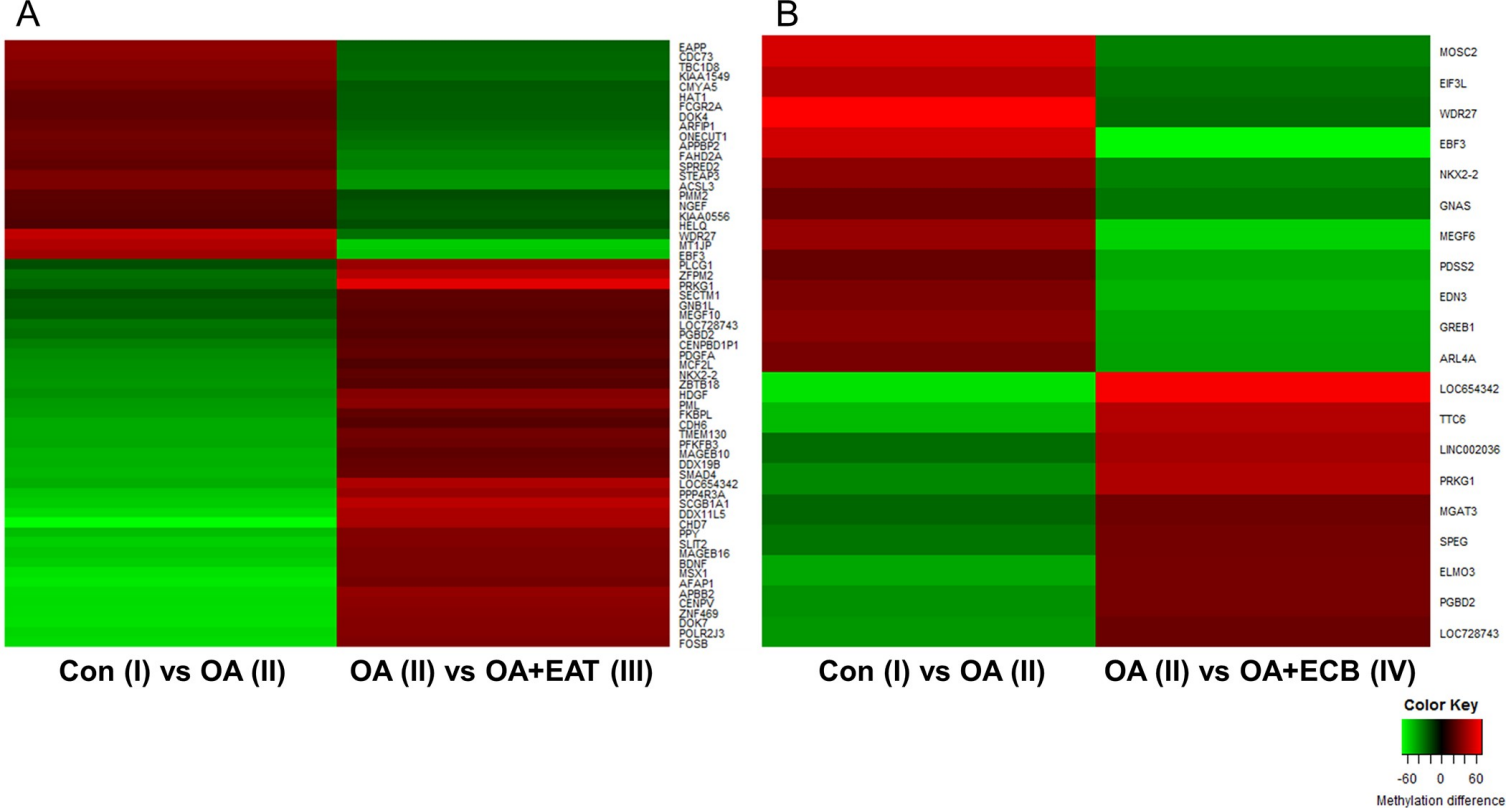
Heatmap of selected DMRs. (A) Heatmap of methylation levels of the 61 DMRs in the control (I) vs OA (II) and OA (II) vs OA+EAT (III) comparisons. (B) 20 DMRs in the control (I) vs OA (II) and OA (II) vs OA+ECB (IV) comparisons (*p* < 0.01, methylation difference between groups >20%).

We further selected 22 DMRs that were hypermethylated in the OA group (II) compared to the control (I), but hypomethylated in the OA+EAT (III) compared to the OA (II), and 39 DMRs showing the converse methylation pattern in the same group comparison (Fig 2A). In addition, 11 DMRs were hypermethylated in the OA (II) but hypomethylated by the OA+ECB (IV), and nine DMRs conversely methylated between the same groups (Fig 2B). A total of 81 regions that were hypermethylated in OA (II) but hypomethylated in OA+EAT (III) and OA+ECB (IV) or vice versa were identified to investigate the effects of EAT and ECB on DNA methylation during hepatic steatosis. As shown in Fig 3, it was evident that the selected DMRs between groups (IIvs I, III vs II, and IV vs II) were clearly clustered and methylation levels were significantly different.

### Selection of putative genes proximally located at the selected DMRs

A total of 77 putative DMRs, excluding regions not assigned by the HOMER program, were identified as regions affected by EAT or ECB in the cell model of hepatic steatosis (Table 4). Among them, 31 DMRs were hypermethylated by OA while 46 DMRs were hypomethylated by OA. The level of DNA methylation in 72 DMRs was reversely changed by treatment with EAT or ECB while only five DMRs were affected by both EAT and ECB. Interestingly, 37 DMRs were located at functional genomic structures such as TSSs, exons, CpG islands, and introns. In view of the known link between hepatic steatosis and the metabolic syndrome [17], we summarized potentially relevant functions of the genes nearest to selected DMRs (Table 4). Among the annotated genes, 26 were found to be related to the metabolic syndrome, including obesity, diabetes, hypertension, cardiovascular diseases, inflammation, and stroke.

**Table 4:** Annotation of selected DMRs.

Position			Gene	DNA methylation at DMR between groups			Genomic region	#CpGs	Association with metabolic syndrome
Chr	Start	End		OA vs Con	OA+EAT vs OA	OA+ECB vs OA			
17	58,564,101	58,564,200	*APPBP2*	Hypermethylation	Hypomethylation	-	LINE	4	NONE
1	3,414,951	3,415,050	*MEGF6*	Hypermethylation	-	Hypomethylation	Exon	8	NONE
7	12,717,651	12,717,800	*ARL4A*	Hypermethylation	-	Hypomethylation	SINE	9	NONE
16	8,941,701	8,941,800	*PMM2*	Hypermethylation	Hypomethylation	-	3’UTR	5	NONE
5	78,985,701	78,985,800	*CMYA5*	Hypermethylation	Hypomethylation	-	TSS	14	Hypertension, cardiomyopathies
16	57,508,751	57,508,850	*DOK4*	Hypermethylation	Hypomethylation	-	Exon	6	Immune response
14	34,992,451	34,992,600	*EAPP*	Hypermethylation	Hypomethylation	-	Intron	9	NONE
10	131,767,451	131,767,600	*EBF3*	Hypermethylation	Hypomethylation	Hypomethylation	CpG	16	NONE
20	57,875,301	57,875,450	*EDN3*	Hypermethylation	-	Hypomethylation	TSS	16	Cardiovascular disease, hypertension, stroke
1	161,432,051	161,432,300	*FCGR2A*	Hypermethylation	Hypomethylation	-	Intergenic	34	Stroke, ulcerative colitis
4	153,788,351	153,788,450	*ARFIP1*	Hypermethylation	Hypomethylation	-	Intron	8	NONE
20	57,465,401	57,465,500	*GNAS*	Hypermethylation	-	Hypomethylation	TSS	16	Hypertension, cardiovascular disease, obesity, diabetes, atherosclerosis
2	11,733,051	11,733,150	*GREB1*	Hypermethylation	-	Hypomethylation	TTS	7	NONE
16	27,781,251	27,781,350	*KIAA0556*	Hypermethylation	Hypomethylation	-	Exon	3	NONE
4	84,320,351	84,320,450	*HELQ*	Hypermethylation	Hypomethylation	-	LINE	4	NONE
2	172,771,151	172,771,250	*HAT1*	Hypermethylation	Hypomethylation	-	Intergenic	2	Asthma
7	138,661,001	138,661,200	*KIAA1549*	Hypermethylation	Hypomethylation	-	Intron	7	NONE
1	220,943,251	220,943,350	*MARC2*	Hypermethylation	-	Hypomethylation	Intron	3	NONE
1	193,109,701	193,109,800	*CDC73*	Hypermethylation	Hypomethylation	-	Intron	3	Hyperlipidemias, myocardial infarction
16	56,669,401	56,669,500	*MT1JP*	Hypermethylation	Hypomethylation	-	TSS	16	NONE
2	233,863,451	233,863,550	*NGEF*	Hypermethylation	Hypomethylation	-	Intron	3	NONE
20	21,503,451	21,503,550	*NKX2-2*	Hypermethylation	-	Hypomethylation	CpG Intergenic	8	Diabetes, obesity
	21,503,151	21,503,300		Hypermethylation	Hypomethylation	-	CpG Intergenic	26	
15	53,079,651	53,079,750	*ONECUT1*	Hypermethylation	Hypomethylation	-	Intergenic	6	Diabetes
6	107,684,801	107,684,900	*PDSS2*	Hypermethylation	-	Hypomethylation	LTR	5	NONE
2	65,928,451	65,928,550	*SPRED2*	Hypermethylation	Hypomethylation	-	Intergenic	5	Arthritis
2	120,000,901	120,001,000	*STEAP3*	Hypermethylation	Hypomethylation	-	Intron TTS	2	NONE
	120,000,951	120,001,050		Hypermethylation					
2	101,747,801	101,747,900	*TBC1D8*	Hypermethylation	Hypomethylation	-	SINE	5	NONE
1	32,696,701	32,696,800	*EIF3I*	Hypermethylation	-	Hypomethylation	TTS	7	NONE
6	169,977,751	169,977,900	*WDR27*	Hypermethylation	Hypomethylation	Hypomethylation	CpG	15	Diabetes
2	223,731,451	223,731,500	*ACSL3*	Hypomethylation	Hypermethylation	-	Intron	3	Hepatic lipogenesis, insulin sensitivity, hepatic steatosis
4	41,218,351	41,218,550	*APBB2*	Hypomethylation	Hypermethylation	-	Intergenic	21	Obesity, diabetes
11	27,722,001	27,722,150	*BDNF*	Hypomethylation	Hypermethylation	-	TSS	20	Obesity, stroke, diabetes, coronary artery disease
22	19,799,851	19,800,000	*GNB1L*	Hypomethylation	Hypermethylation	-	Exon	7	NONE
17	80,278,851	80,279,000	*SECTM1*	Hypomethylation	Hypermethylation	-	TTS	14	NONE
5	30,346,051	30,346,200	*CDH6*	Hypomethylation	Hypermethylation	-	LTR	11	NONE
19	59,093,201	59,093,300	*CENPBD1P1*	Hypomethylation	Hypermethylation	-	Non-coding	8	NONE
17	16,258,051	16,258,150	*CENPV*	Hypomethylation	Hypermethylation	-	Simple repeats	5	NONE
8	61,626,901	61,627,050	*CHD7*	Hypomethylation	Hypermethylation	-	CpG	11	NONE
9	11,101	11,250	*DDX11L5*	Hypomethylation	Hypermethylation	-	TSS	19	NONE
16	70,323,601	70,323,750	*DDX19B*	Hypomethylation	Hypermethylation	-	TSS	9	NONE
4	3,465,101	3,465,250	*DOK7*	Hypomethylation	Hypermethylation	-	CpG	19	Lipid metabolism
18	48,533,301	48,533,400	*ELAC1* */SMAD4*	Hypomethylation	Hypermethylation	-	LTR	3	Cardiovascular disease, hypertension, diabetes
2	96,066,001	96,066,100	*FAHD2A*	Hypomethylation	Hypermethylation	-	LTR	4	NONE
6	32,099,051	32,099,150	*FKBPL*	Hypomethylation	Hypermethylation	-	Intergenic	9	Angiogenesis
15	74,340,851	74,341,000	*PML*	Hypomethylation	Hypermethylation	-	TTS	8	Hypertension, stroke, coronary artery disease
1	156,717,001	156,717,100	*HDGF*	Hypomethylation	Hypermethylation	-	Simple repeats	6	Hypertension
3	193,922,151	193,922,250	*LINC002036*	Hypomethylation	-	Hypermethylation	CpG	14	NONE
4	7,864,101	7,864,200	*AFAP1*	Hypomethylation	Hypermethylation	-	Intron	5	NONE
2	91,634,801	91,634,950	*LOC654342*	Hypomethylation	Hypermethylation	Hypermethylation	CpG	19	NONE
7	150,105,001	150,105,100	*LOC728743*	Hypomethylation	Hypermethylation	-	Non-coding	21	NONE
X	27,827,551	27,827,700	*MAGEB10*	Hypomethylation	Hypermethylation	-	LTR	18	NONE
X	35,517,551	35,517,700	*MAGEB16*	Hypomethylation	Hypermethylation	-	LTR	13	NONE
13	113,705,001	113,705,100	*MCF2L*	Hypomethylation	Hypermethylation	-	Intron	10	Cardiovascular disease, atherosclerosis
5	126,626,501	126,626,600	*MEGF10*	Hypomethylation	Hypermethylation	-	TSS	8	NONE
22	39,853,201	39,853,300	*MGAT3*	Hypomethylation	-	Hypermethylation	TSS	22	NONE
16	67,235,901	67,236,050	*ELMO3*	Hypomethylation	-	Hypermethylation	TTS	9	NONE
19	45,954,101	45,954,250	*FOSB*	Hypomethylation	Hypermethylation	-	Intergenic	22	NONE
20	39,795,151	39,795,250	*PLCG1*	Hypomethylation	Hypermethylation	-	Exon	5	NONE
4	4,858,701	4,858,850	*MSX1*	Hypomethylation	Hypermethylation	-	Intergenic	15	NONE
7	559,501	559,600	*PDGFA*	Hypomethylaton	Hypermethylation	-	TSS	12	Asthma
10	6,242,601	6,242,700	*PFKFB3*	Hypomethylation	Hypermethylation	-	Simple repeats	3	Insulin resistance, diabetes, obesity, inflammation
1	249,239,551	249,239,700	*PGBD2*	Hypomethylation	Hypermethylation	Hypermethylation	Intergenic	7	NONE
7	102,213,151	102,213,250	*POLR2J3*	Hypomethylation	Hypermethylation	-	TSS	11	NONE
14	92,044,551	92,044,650	*CATSPERB* */SMEK1*	Hypomethylation	Hypermethylation	-	LTR	3	NONE
17	42,015,751	42,015,900	*PPY*	Hypomethylation	Hypermethylation	-	Simple repeats	12	Diabetes, obesity
10	52,834,351	52,834,450	*PRKG1*	Hypomethylation	Hypermethylation	Hypermethylation	Exon	6	Cardiovascular disease, hypertension, stroke, diabetes
11	62,192,201	62,192,350	*SCGB1A1*	Hypomethylation	Hypermethylation	-	Intergenic	9	Hypertension, diabetes, stroke, asthma
4	19,415,401	19,415,550	*SLIT2*	Hypomethylation	Hypermethylation	-	Simple repeats	8	Hypertension, diabetes, obesity, stroke
2	220,313,351	220,313,450	*SPEG*	Hypomethylation	-	Hypermethylation	Exon	15	Cardiovascular disease
7	98,384,201	98,384,350	*TMEM130*	Hypomethylation	Hypermethylation	-	LTR	4	NONE
14	38,067,501	38,067,550	*TTC6*	Hypomethylation	-	Hypermethylation	CpG	3	NONE
7	150,105,051	150,105,150	*LOC728743*	Hypomethylation		Hypermethylation	non-coding	15	NONE
1	244,354,201	244,354,300	*ZBTB18*	Hypomethylation	Hypermethylation	-	Ingergenic	3	NONE
8	106,330,701	106,330,850	*ZFPM2*	Hypomethylation	Hypermethylation	-	TSS	20	NONE
16	88,476,201	88,476,350	*ZNF469*	Hypomethylation	Hypermethylation	-	Intergenic	7	NONE

### Gene expression affected by modulation of DNA methylation by EAT and ECB

**Fig 4:**
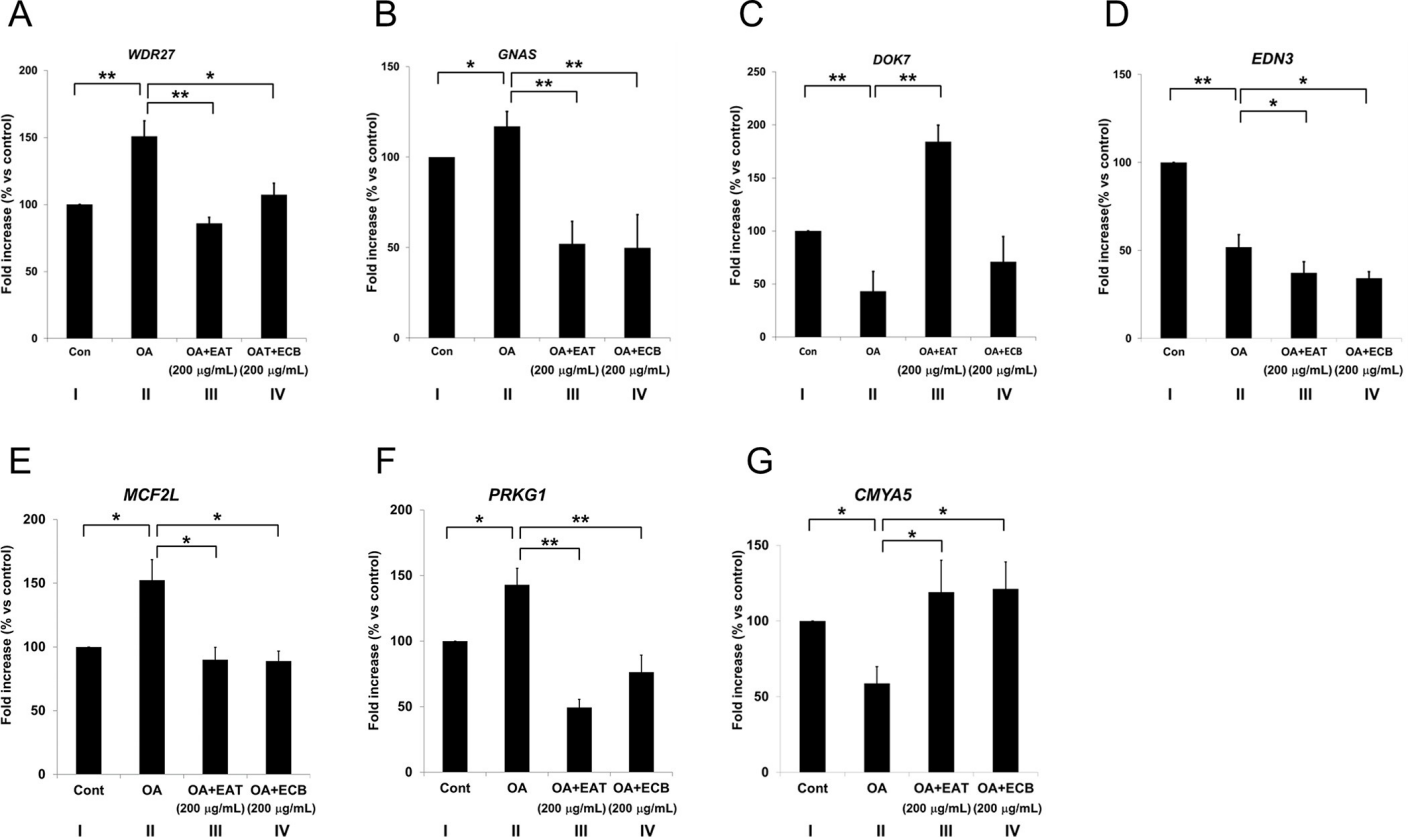
Expression of putative genes proximal to DMRs. Expression levels of genes differentially methylated by EAT or ECB. After HepG2 cells were stimulated with 0.5 mM OA (II), OA with EAT (200 μg/mL, III), and OA with ECB (200 μg/mL, IV) for 24 h, expression of candidate genes identified from RRBS analysis was examined with RT-qPCR. Data was shown as mean ± SD (n = 4, *WDR27*, *GNAS*, *DOK7*, *EDN3*; n = 7, *MCF2L*, *PRKG1*, *CMYA5*). ** *p* < 0.01, * *p* < 0.05.

To investigate the effects of DNA methylation on expression of genes proximal to DMRs, we examined the association of expression levels of genes with the selected DMRs in hepatic steatosis. As shown in Fig 4, seven genes (*WDR27*, *GNAS*, *DOK7*, *EDN3*, *MCF2L*, *PRKG1*, and *CMYA5*) were selected based on genomic location and relevance to metabolic syndrome. Expression of *WDR27*, *GNAS*, *MCF2L*, and *PRKG1* was increased by OA but decreased by treatment with EAT or ECB (Fig 4A, 4B, 4E and 4F). Expression of *DOK7* and *CMYA5* was decreased by OA but increased by EAT (Fig 4C and 4G). Expression of *EDN3* was decreased by OA but not changed by EAT or ECB (Fig 4D). DMR location of each gene was marked as thick red line in S3 Fig. Methylation levels of the DMR were shown in S3 Table.

## Discussion

### Selection of DMRs associated with hepatic steatosis

More than 60% of our selected DMRs were located at functional genomic regions such as TSSs, exons, 5’ UTRs, CpG islands, and introns, while less than 40% of the non-selected DMRs were located in these regions (Table 3). This suggested that the selected DMRs may be more likely to be involved in regulation of genes expression.

The number of hypermethylated DMRs was not substantially different from that of hypomethylated DMRs in the analysis of OA (II) vs control (I), or in our other comparisons (III vs II and IV vs II) (Table 4). This suggests that both up- and down-regulation of DNA methylation are involved in lipid accumulation and may stimulate or suppress gene expression, and is consistent with reports showing that some genes (*FASN*, *PPARγ*, and *SREBP1*) are increased but others (*SIRT1*, *FOXO1*, and *ATGL*) are decreased in cell models of hepatic steatosis [24, 33].

### Effects of dietary components on DNA methylation

*Allium tuberosum* (AT) and *Capsella bursa-pastoris* (CB) have been widely consumed as food ingredients in Korea. It has been known that AT exerts various health benefits in inflammation, diabetes, and cardiovascular diseases, as does CB in inflammation and cancer [34, 35]. However, their underlying mechanisms are not fully understood. Recently, it was suggested that histone modifications by EAT and ECB may be involved in alleviating hepatic steatosis and provide a therapeutic target for its treatment or prevention [23, 24]. This study showed for the first time that EAT and/or ECB reversed DNA methylation induced by OA in an in vitro cell model of hepatic steatosis (Table 4).

Many studies demonstrated that *Allium tubersosum* (AT) and *Capsella bursa-pastoris* (CB) consist of sulphur-containing compounds, phenolic compounds, acylated flavonol glucosides, flavonoids, organic acid, and other many compounds [36–39]. Among these compounds, both AT and CB contain same flavonoid compounds such as kaempferol and quercetin. Kaempferol and quercetin are flavonoid compounds having an antioxidant activity. It has been known that they improved NAFLD by reducing hepatic lipid accumulation and oxidative stress [40–42]. It was also reported that kaempferol and quercetin induced epigenetic modifications through regulating histone deacetylases (HDACs) and/or DNMTs [43–45]. These suggested that AT and CB may improve NAFLD by regulating DNA methylation.

### Effects of dietary component on selected genes involved in hepatic steatosis

We investigated the correlation of DNA methylation with expression of seven genes (*WDR27*, *GNAS*, *DOK7*, *EDN3*, *CMYA5*, *PRKG1*, and *MCF2L*) selected on the basis of their known functions in metabolic syndrome [46–54], and their locations in functional genomic regions.

It is generally known that hypermethylated DNA suppresses gene expression while hypomethylation stimulates transcription. Consistent with it, we showed hypomethylation at an intron of *MCF2L* and an exon of *PRKG1* by OA (Table 4), and the hypomethylatoin was associated with increased expression of the genes (Fig 4E and 4F). EAT and ECB induced hypermethylation of *MCF2L* and *PRKG1* and decreased their expression. In addition, hypermethylation at transcription start site of *CMYA5* by OA decreased its expression while hypomethylated by EAT increased its expression (Table 4 and Fig 4G). These suggest that level of methylation of *MCF2L*, *PRKG1*, and *CMYA5* may regulate expression of the genes.

Although the physiological function of *WDR27* has not been fully demonstrated, an SNP in intergenic region adjoining *WDR27* (rs924043) was associated with type 1 diabetes, which suggests that its expression may be involved in metabolic syndrome [46]. In addition, duplication of *WDR27* has been seen in an obese patient, which suggests that *WDR27* may be overexpressed in obesity [47]. Consistent with this, *WDR27* its expression was significantly increased by OA while decreased by EAT and ECB (Fig 4A). In this study, we showed that an intron of *WDR27* was hypermethylated in the OA group but hypomethylated after treatment with EAT and ECB (Table 4). It is important to note that DNA hypermethylation can increase expression of genes although it is generally known that hypermethylation suppresses gene expression. Recently, this was supported by a systematic analysis of binding of 542 transcription factors (TFs) to methylated or unmethylated CpGs [48]. For activation of gene expression by the TFs, 34% and 23% of the TFs preferred hypermethylated and hypomethylated CpGs respectively, while 33% of the TFs did not prefer CpGs. Together, this suggested that DNA hypermethylation can also stimulate gene expression.

It is known that *GNAS* regulates homeostasis of glucose and energy metabolism [49]. Interestingly, the methylation level of CpG sites located at the upstream of the *GNAS* TSS was significantly decreased after dietary intervention [50]. Consistent with this, we showed that this TSS region was hypomethylated by ECB. Significantly decreased gene expression was also observed with ECB treatment (Fig 4B). Together, this suggested that DMRs in the *GNAS* TSS region were affected by dietary factors and associated with its transcription.

It was reported that *DOK7* plays a crucial role in the progress of metabolic disease in an animal model through regulation of DNA methylation at its promoter, affecting its expression [51]. We found that exonic and intronic CpG islands in *DOK7* were hypomethylated by OA and hypermethylated by EAT (Table 4), and that expression of the gene was decreased by OA and elevated by EAT in our cell model of hepatic steatosis (Fig 4C). Together, these findings also suggest that DNA methylation at CpG islands in *DOK7* are regulated by dietary factors and associated with its expression.

Although it is known that genetic variants in a region between *GNAS* and *EDN3* are associated with hypertension and cardiovascular disease [52, 53], DNA methylation may not involve in expression of *EDN3* in cell model of hepatic steatosis since hypermethylation and hypomethylation at the TSS of *EDN3* by OA and ECB, respectively (Table 4), decreased expression of *EDN3* (Fig 4D).

Although this study showed that hepatic steatosis in cell model was affected by DNA methylation regulating expression of each gene by dietary factors, it did not exclude the possibility that the selected genes may synergistically play roles in hepatic steatosis. As previously described, NAFLD is caused by multi-factors such as SREBP-1c, PPARγ, and FASN. It was also demonstrated that other factors and different regulatory mechanisms were involved in the progression of hepatic steatosis [54, 55, 17]. These studies suggest that several factors instead of a factor may synergistically and/or spatiotemporally play roles during hepatic steatosis. Further study will be required to uncover whether all the selected genes exert their functions in a combinational manner during hepatic steatosis.

Since cell line systems do not reflect exact whole organisms such as interactions with other cell types/tissues, metabolic status and effect of hormones etc., it has been still controversial whether the significance of cell line data can be reproduced in *in vivo* studies. However, cell line systems are very efficient to select or narrow down targets through screening of compounds and will provide information for further studies. This study described regulation of DMRs during steatosis in a cell model and will help further investigate the animal or clinical studies.

In conclusion, this study showed, for the first time, that modulation of DNA methylation is one of the mechanisms during hepatic steatosis in a cell model. This study also showed the regulation of expression of genes by DNA methylation in hepatic steatosis model alleviated by EAT and ECB. The data present here provide a potential lead into further studies investigating hepatic steatosis and may give an insight to development of prevention or treatment of hepatic steatosis.

## Supporting information

S1 Table List of primers for qRT-PCR. (PPTX)

S2 Table Mapping summary of RRBS. (PPTX)

S3 Table Methylation level of selected DMRs. (PPTX)

S1 Fig Cytotoxicity of EAT and ECB in HepG2 cells. HepG2 cells were treated with different concentration of ECB or EAT in the absence of OA for 24h. Cell cytotoxicity was determined. Data are expressed as mean ± SD (n = 3).
(TIF)

S2 Fig Expression of FASN in HepG2 cells treated with EAT and ECB. HepG2 cells were stimulated with OA, OA with EAT (200 μg/mL), OA with ECB (200 μg/mL). Expression levels of general lipid metabolism markers (FASN) and β-actin control protein level were assayed by Western blot.
(TIF)

S3 Fig Schematic illustration of DMR location of the selected genes. The genomic diagram was obtained from UCSC Genome Browsers (http://genome.uscs.edu). DMR location of each gene was marked as thick red line. DMRs of *WDR27* and *DOK7* were located at CpG islands annotated by HOMER. (A) *WDR27*, (B) *GNAS*, (C) *DOK7*, (D) *EDN3*, (E) *MCF2L*, (F) *PRKG1*, (G) *CMYA5*.
(TIF)
