# Supplementary material for: Genome-wide analysis of DNA methylation identifies novel differentially methylated regions associated with lipid accumulation improved by ethanol extracts of Allium tubersosum and Capsella bursa-pastoris in a cell model
Source: PLoS One. 2019 Jun 6;14(6):e0217877. doi: 10.1371/journal.pone.0217877 (PMC6553759; doi:10.1371/journal.pone.0217877)
Supplement: S1 Table — (PPTX) [file pone.0217877.s001.pptx]

## Slide 1
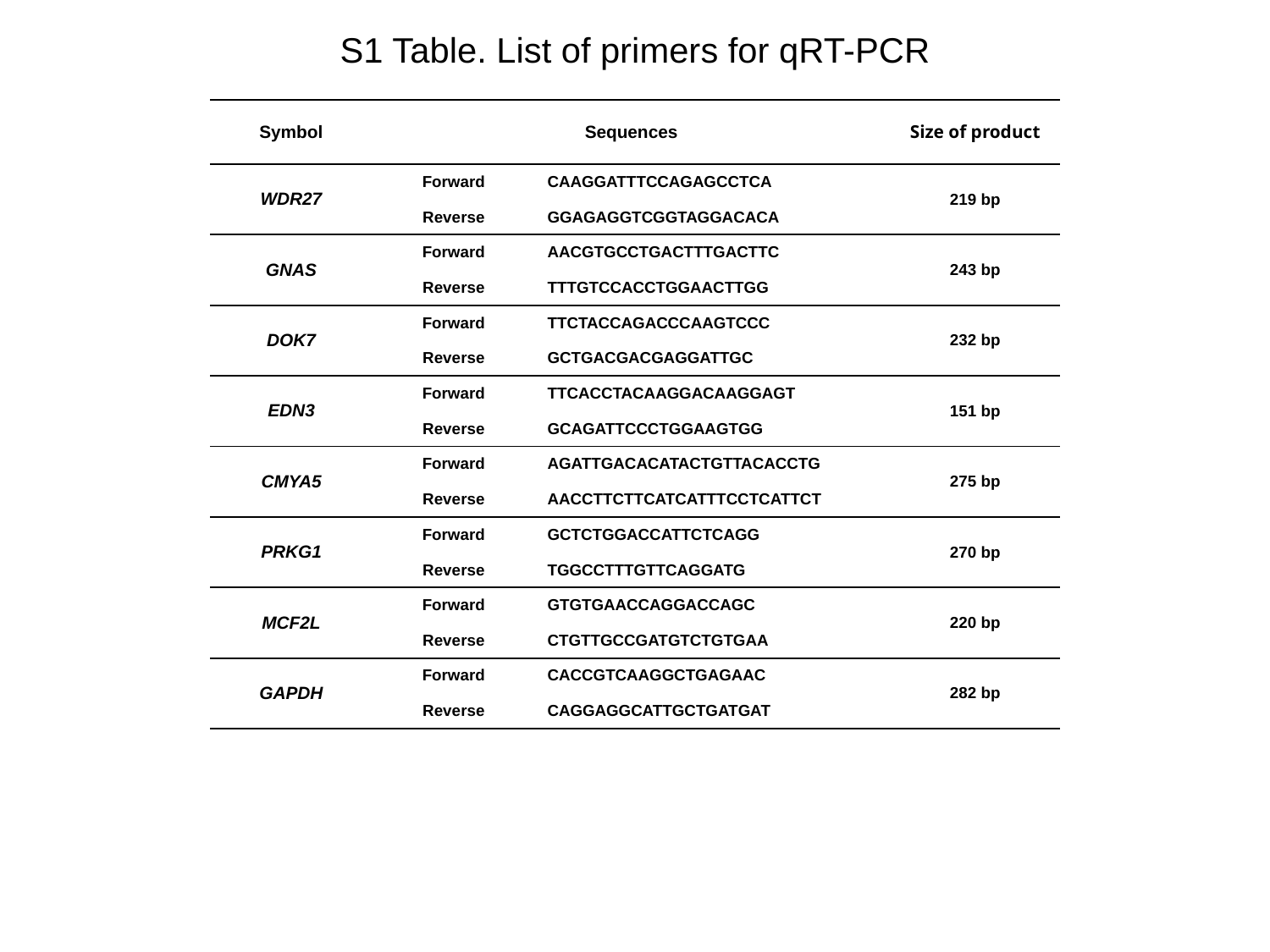

S1 Table. List of primers for qRT-PCR
| Symbol | Sequences | | Size of product |
| --- | --- | --- | --- |
| WDR27 | Forward | CAAGGATTTCCAGAGCCTCA | 219 bp |
| | Reverse | GGAGAGGTCGGTAGGACACA | |
| GNAS | Forward | AACGTGCCTGACTTTGACTTC | 243 bp |
| | Reverse | TTTGTCCACCTGGAACTTGG | |
| DOK7 | Forward | TTCTACCAGACCCAAGTCCC | 232 bp |
| | Reverse | GCTGACGACGAGGATTGC | |
| EDN3 | Forward | TTCACCTACAAGGACAAGGAGT | 151 bp |
| | Reverse | GCAGATTCCCTGGAAGTGG | |
| CMYA5 | Forward | AGATTGACACATACTGTTACACCTG | 275 bp |
| | Reverse | AACCTTCTTCATCATTTCCTCATTCT | |
| PRKG1 | Forward | GCTCTGGACCATTCTCAGG | 270 bp |
| | Reverse | TGGCCTTTGTTCAGGATG | |
| MCF2L | Forward | GTGTGAACCAGGACCAGC | 220 bp |
| | Reverse | CTGTTGCCGATGTCTGTGAA | |
| GAPDH | Forward | CACCGTCAAGGCTGAGAAC | 282 bp |
| | Reverse | CAGGAGGCATTGCTGATGAT | |
