# Supplementary material for: Genome-wide analysis of DNA methylation identifies novel differentially methylated regions associated with lipid accumulation improved by ethanol extracts of Allium tubersosum and Capsella bursa-pastoris in a cell model
Source: PLoS One. 2019 Jun 6;14(6):e0217877. doi: 10.1371/journal.pone.0217877 (PMC6553759; doi:10.1371/journal.pone.0217877)
Supplement: S2 Table — (PPTX) [file pone.0217877.s002.pptx]

## Slide 1
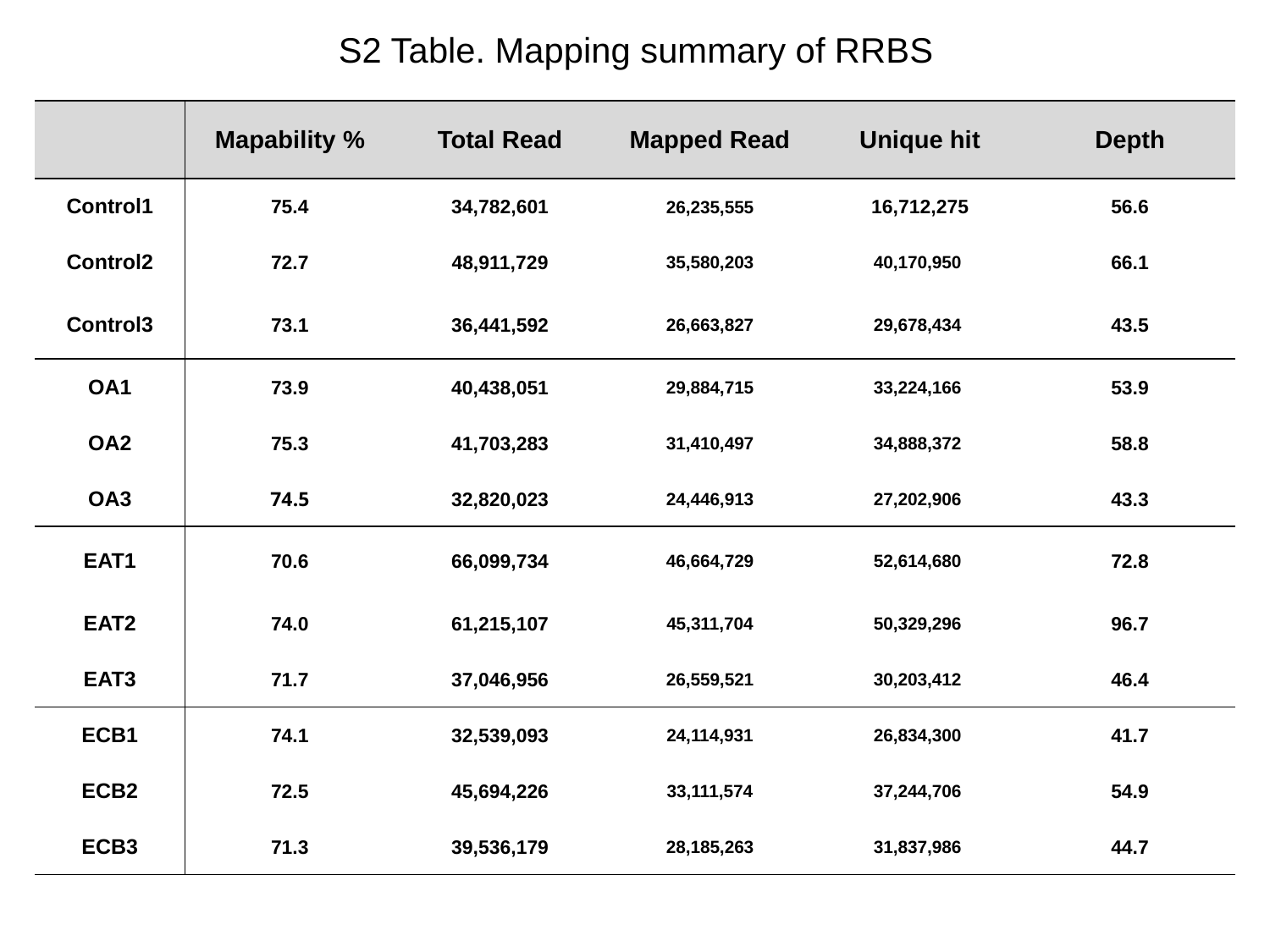

S2 Table. Mapping summary of RRBS
| | Mapability % | Total Read | Mapped Read | Unique hit | Depth |
| --- | --- | --- | --- | --- | --- |
| Control1 | 75.4 | 34,782,601 | 26,235,555 | 16,712,275 | 56.6 |
| Control2 | 72.7 | 48,911,729 | 35,580,203 | 40,170,950 | 66.1 |
| Control3 | 73.1 | 36,441,592 | 26,663,827 | 29,678,434 | 43.5 |
| OA1 | 73.9 | 40,438,051 | 29,884,715 | 33,224,166 | 53.9 |
| OA2 | 75.3 | 41,703,283 | 31,410,497 | 34,888,372 | 58.8 |
| OA3 | 74.5 | 32,820,023 | 24,446,913 | 27,202,906 | 43.3 |
| EAT1 | 70.6 | 66,099,734 | 46,664,729 | 52,614,680 | 72.8 |
| EAT2 | 74.0 | 61,215,107 | 45,311,704 | 50,329,296 | 96.7 |
| EAT3 | 71.7 | 37,046,956 | 26,559,521 | 30,203,412 | 46.4 |
| ECB1 | 74.1 | 32,539,093 | 24,114,931 | 26,834,300 | 41.7 |
| ECB2 | 72.5 | 45,694,226 | 33,111,574 | 37,244,706 | 54.9 |
| ECB3 | 71.3 | 39,536,179 | 28,185,263 | 31,837,986 | 44.7 |
