# Supplementary material for: Genome-wide analysis of DNA methylation identifies novel differentially methylated regions associated with lipid accumulation improved by ethanol extracts of Allium tubersosum and Capsella bursa-pastoris in a cell model
Source: PLoS One. 2019 Jun 6;14(6):e0217877. doi: 10.1371/journal.pone.0217877 (PMC6553759; doi:10.1371/journal.pone.0217877)
Supplement: S3 Table — (PPTX) [file pone.0217877.s003.pptx]

## Slide 1
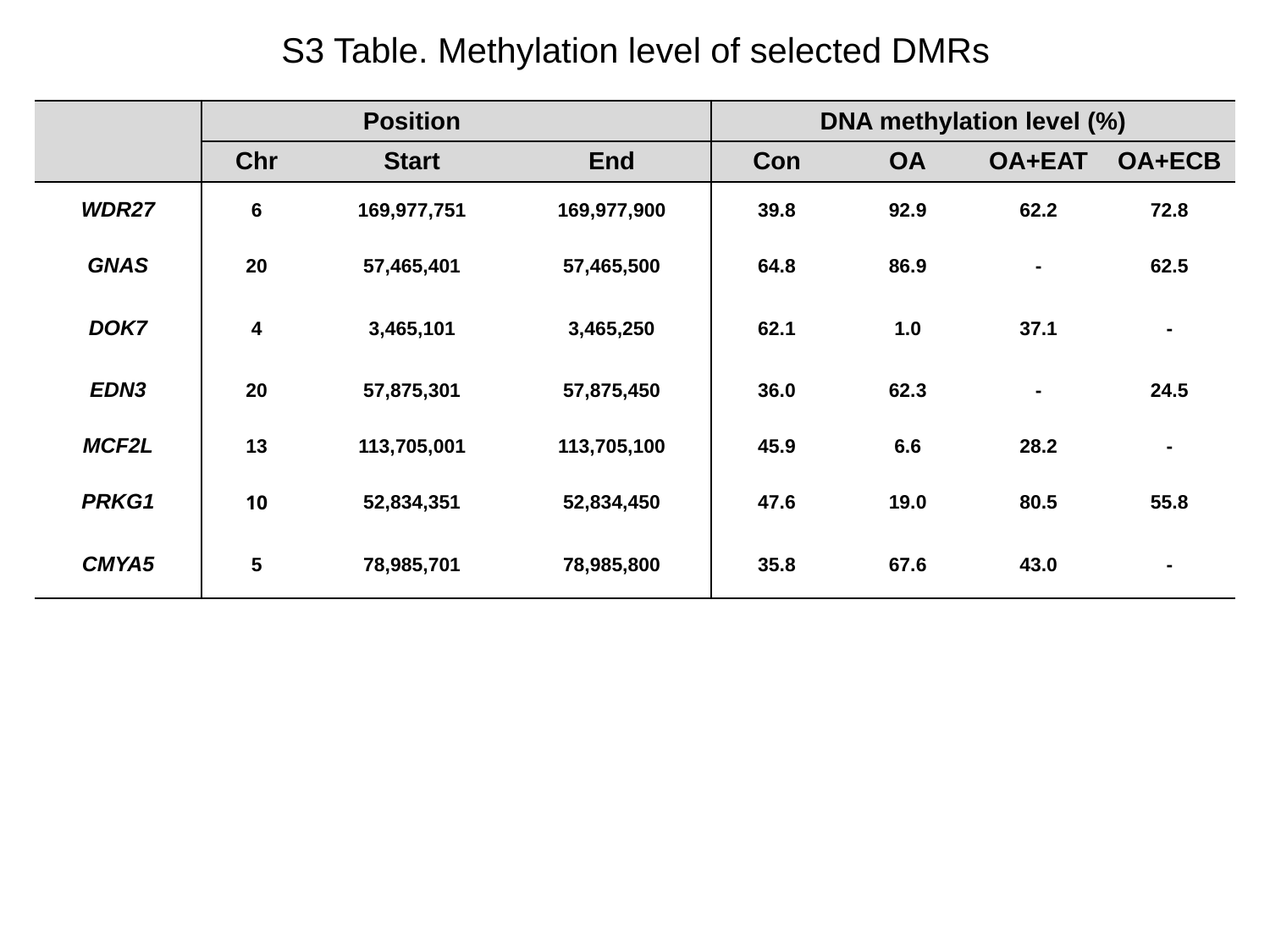

S3 Table. Methylation level of selected DMRs
| | | Position | | DNA methylation level (%) | | | |
| --- | --- | --- | --- | --- | --- | --- | --- |
| | Chr | Start | End | Con | OA | OA+EAT | OA+ECB |
| WDR27 | 6 | 169,977,751 | 169,977,900 | 39.8 | 92.9 | 62.2 | 72.8 |
| GNAS | 20 | 57,465,401 | 57,465,500 | 64.8 | 86.9 | - | 62.5 |
| DOK7 | 4 | 3,465,101 | 3,465,250 | 62.1 | 1.0 | 37.1 | - |
| EDN3 | 20 | 57,875,301 | 57,875,450 | 36.0 | 62.3 | - | 24.5 |
| MCF2L | 13 | 113,705,001 | 113,705,100 | 45.9 | 6.6 | 28.2 | - |
| PRKG1 | 10 | 52,834,351 | 52,834,450 | 47.6 | 19.0 | 80.5 | 55.8 |
| CMYA5 | 5 | 78,985,701 | 78,985,800 | 35.8 | 67.6 | 43.0 | - |
