# Supplementary figures and images for: Genome-wide analysis of DNA methylation identifies novel differentially methylated regions associated with lipid accumulation improved by ethanol extracts of Allium tubersosum and Capsella bursa-pastoris in a cell model
Source: PLoS One. 2019 Jun 6;14(6):e0217877. doi: 10.1371/journal.pone.0217877 (PMC6553759; doi:10.1371/journal.pone.0217877)

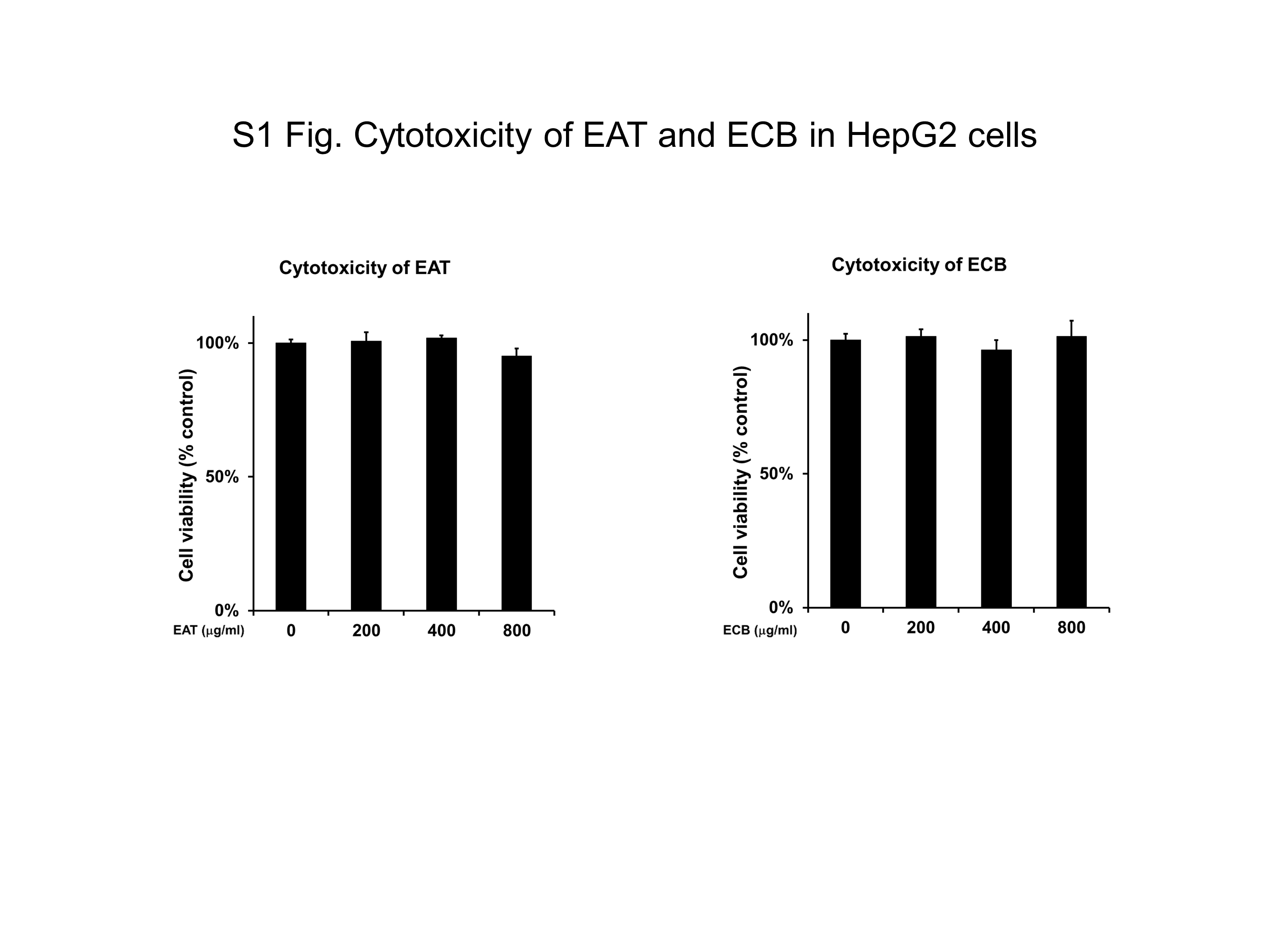

Supplement: S1 Fig — HepG2 cells were treated with different concentration of ECB or EAT in the absence of OA for 24h. Cell cytotoxicity was determined. Data are expressed as mean ± SD (n = 3). (TIF) [file pone.0217877.s004.tif]

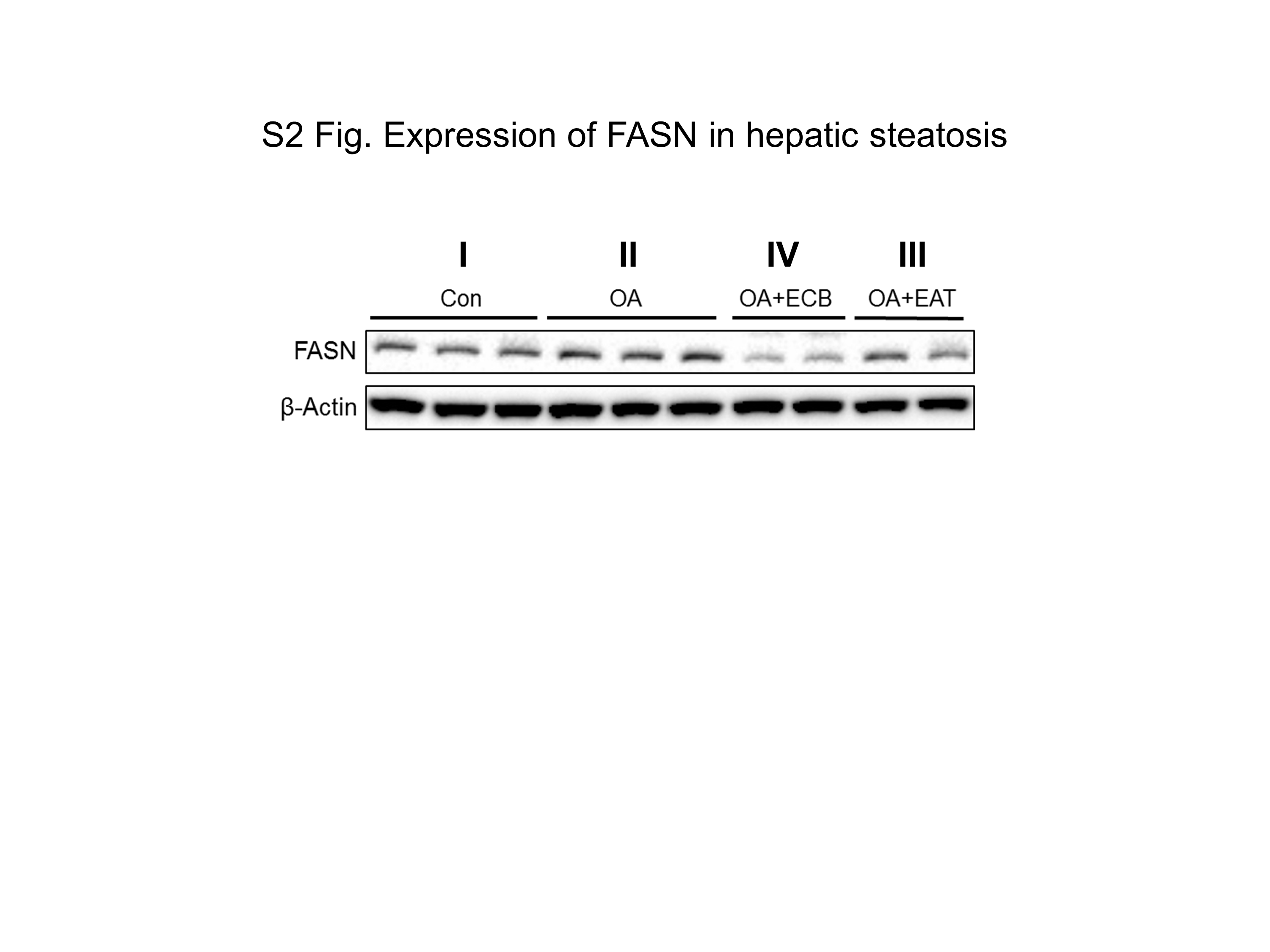

Supplement: S2 Fig — HepG2 cells were stimulated with OA, OA with EAT (200 μg/mL), OA with ECB (200 μg/mL). Expression levels of general lipid metabolism markers (FASN) and β-actin control protein level were assayed by Western blot. (TIF) [file pone.0217877.s005.tif]

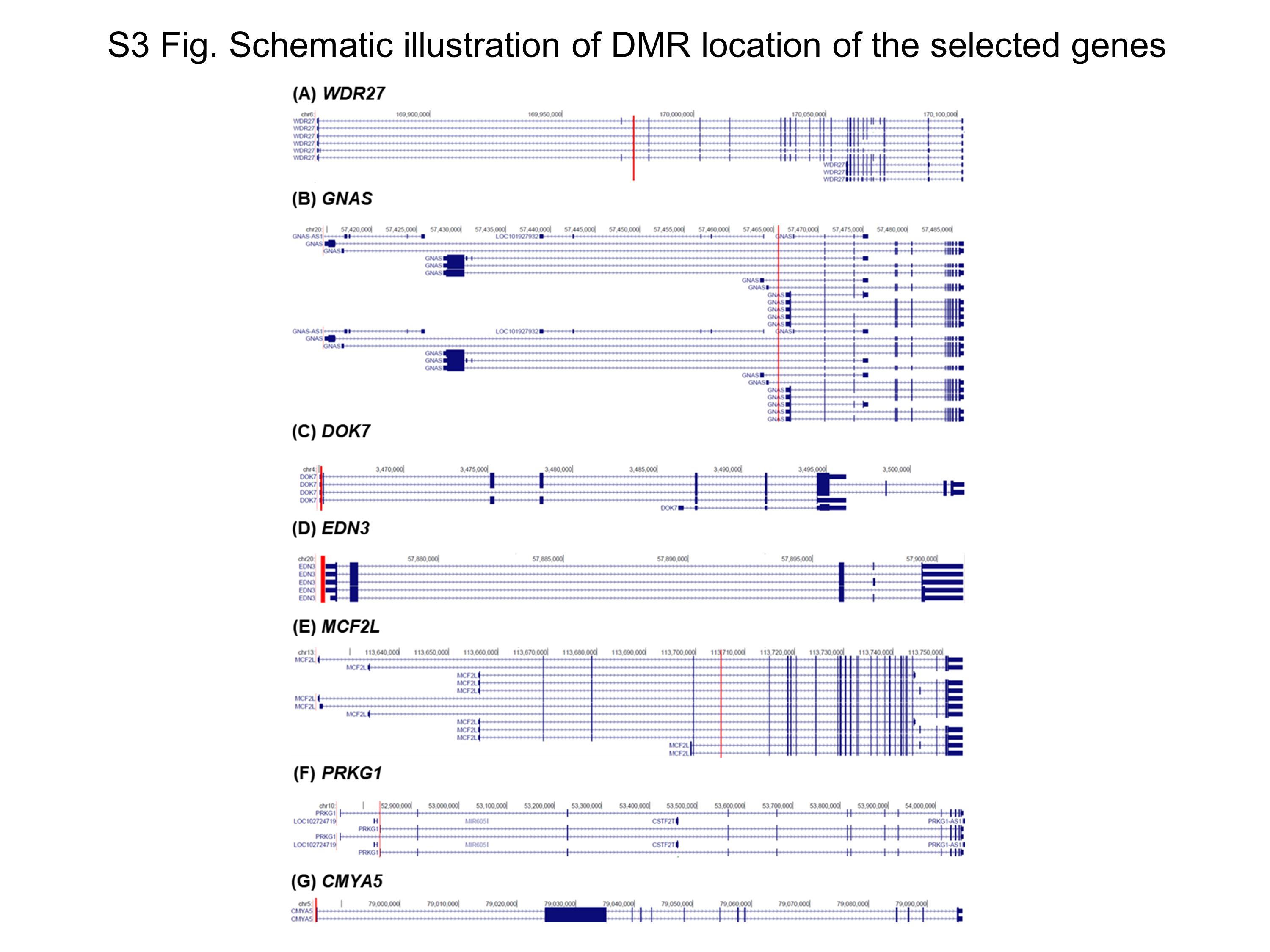

Supplement: S3 Fig — The genomic diagram was obtained from UCSC Genome Browsers (http://genome.uscs.edu). DMR location of each gene was marked as thick red line. DMRs of WDR27 and DOK7 were located at CpG islands annotated by HOMER. (A) WDR27, (B) GNAS, (C) DOK7, (D) EDN3, (E) MCF2L, (F) PRKG1, (G) CMYA5. (TIF) [file pone.0217877.s006.tif]
